# Engaging diverse populations in aging research during the COVID-19 pandemic: Lessons learned from four National Institutes of Health funded-Centers

**DOI:** 10.3389/fpubh.2023.1062385

**Published:** 2023-04-03

**Authors:** Irving E. Vega, Kristine J. Ajrouch, Vanessa Rorai, Renee Gadwa, J. Scott Roberts, Linda Nyquist

**Affiliations:** ^1^Michigan Center for Contextual Factors in Alzheimer’s Disease, Ann Arbor, MI, United States; ^2^Department of Translational Neuroscience, College of Human Medicine, Michigan State University, East Lansing, MI, United States; ^3^Department of Sociology, Anthropology, and Criminology, Eastern Michigan University, Ypsilanti, MI, United States; ^4^Institute for Social Research, University of Michigan, Ann Arbor, MI, United States; ^5^Michigan Center for Urban African American Aging Research, Detroit, MI, United States; ^6^Institute of Gerontology, Wayne State University, Detroit, MI, United States; ^7^Outreach, Recruitment and Engagement Core, Michigan Alzheimer’s Disease Research Center, Ann Arbor, MI, United States; ^8^Department of Health Behavior and Health Education, University of Michigan School of Public Health, Ann Arbor, MI, United States; ^9^Claude D. Pepper Older Americans Independence Center, University of Michigan, Ann Arbor, MI, United States

**Keywords:** aging, community engagement, underrepresented groups, COVID-19, centers and institutes

## Abstract

**Background:**

The COVID-19 pandemic’s impact on our personal and professional lives required a rapid adaptation to the evolving health crisis and accumulating social stresses. Established measures to reduce the spread of infection and potential death had a direct effect on ongoing research that involved older adults and underrepresented racial/ethnic groups. Although important to preserve public health, these measures risk further isolation of vulnerable research participant populations and threatened established community partnerships. To address the social and research challenges evolving from the COVID-19 pandemic, four National Institutes of Health funded-Centers that engage with community members to enhance research and advance the science of aging came together to learn from each other’s efforts, approaches, and communication with community partners.

**Methods:**

Monthly meetings served as a venue to discuss the challenges of engagement with research participants and support community partners during the pandemic. The developed learning community also contributed to recognize and address research staff stress and isolation. We describe how these conversations led our Centers to address unprecedented challenges and sustain community engagement within diverse populations, especially Black/African Americans, Latinos, Middle Eastern/Arab Americans and the oldest-old.

**Results:**

The exchange of information resulted in maintaining long standing community relationships and partnerships in the face of the uncertainties generated by the pandemic. The strategies included adapting education programs to reduce risk of infection, recognizing symptoms, promoting vaccination and understanding of the effect of COVID-19 to the brain. Different strategies were used to address the effects of isolation and maintain community engagement. Although new research participant enrollment was a challenge, telephone and virtual visits allowed research participants to remain active in research. Community members participation in virtual learning events was variable, ranging from a dozen to hundreds of participants. Invitations to organize panels about newly developed topics indicated the need for information from trusted sources.

**Conclusion:**

In sum, the COVID-19 pandemic re - directed all four Centers’ commitment to community service led to developing strategies for social support, which will potentially contribute to transforming public perceptions about research and researchers.

## Introduction

Several reports highlighted the importance of effective implementation of community engagement strategies, especially among most vulnerable and disproportionately affected groups, during the COVID-19 pandemic (1–7). Community engagement is defined as an intentional effort to promote participative decision-making and governance with specific groups to establish relationships and enhance trust between organizations and communities (2). The communication between community members and academic researchers or government officials was the common factor to maintain community engagement during the COVID-19 pandemic throughout the world (1–7). Leverage of an established community advisory committee or community coalition board was identified as an effective way to gather information about community needs, health concerns as well as collective beliefs regarding the evolving pandemic and policies (2–5). This co-leadership and equitable partnership served to develop education programming and advocacy strategies to align resources that address and mitigate the identified community needs (3). Importantly, the established relationship allowed researchers and health professionals to be responsive and sensitive to cultural, traditions and collective beliefs within the community, which guided the effective use of modern and traditional media outlets and public communication strategies. These became even more relevant to address the restrictions on academic research during the COVID-19 pandemic that restricted in-person visits and required the transition to the virtual space, especially when involving the oldest old in the population.

Those involved in community engagement to enhance research participation of underserved groups and advance knowledge about aging faced new challenges in sustainable community engagement during, and after the COVID-19 pandemic. Specifically, COVID-19 disproportionately affected the underrepresented and underserved sectors of our population (8–14) including African American/Blacks as well as Middle Eastern/Arab Americans in metro-Detroit and Latinos and older adults in West Michigan (15). From higher rates of infection to increased food and housing insecurity, the pandemic unearthed the vulnerabilities and inequities that affect underserved communities (8–15). The implemented social distancing and mobility policies, restrictions and suspension of research protocols that involved human participants, and work from home rules had the potential to strain the relationships built over years with community members and research participants. The disruption of established partnerships with grassroot community organizations and the exacerbated needs within the African American/Black, Latinos and Middle Eastern/Arab American communities represented an unprecedented challenge that put in jeopardy the established relationships with community organizations and the community at-large.

Throughout the first year of the COVID-19 pandemic, the social distancing measures led to university campus shutdown and reduced research activities, which had a direct impact on research that involved human participants and community engagement. Four National Institutes of Health (NIH) funded-Centers in Michigan, each with a main objective to engage community members to enhance research participation of underrepresented groups and advance the science of aging, had to adapt their activities. The four National Institutes of Health funded-Centers are: Michigan Center for Contextual Factor in Alzheimer’s disease (MCCFAD), Michigan Center for Urban African American Aging Research (MCUAAAR), Michigan Alzheimer’s Disease Research Center (Michigan ADRC) and University of Michigan Claude D. Pepper Older Americans Independence Center.

MCCFAD is a National Institute on Aging Alzheimer’s disease Resource Center for Minority Aging Research (AD-RCMAR), funded in 2018. MCCFAD focuses on generating knowledge concerning the contexts of Alzheimer’s disease and related dementias (ADRD) by elucidating sociocultural, economic and behavioral contributors to and consequences of health disparities, specifically among the Middle Eastern/North African/Arab Americans in the metropolitan Detroit area and Latino communities in the Grand Rapids Metropolitan area. MCCFAD’s Community Liaison and Recruitment Core (CLRC) uses a community-based partnership research approach to develop and disseminate culturally tailored health education programs to reduce risk of ADRD and enhance participation in the healthcare system (16). Another goal is to encourage research participation by inviting community members to become part of a Research Volunteer Directory (RVD). In the last 4 years, we enrolled over 800 Middle Eastern/North African/Arab Americans and Latinos in the RVD.

Founded in 1998, MCUAAAR is a pioneering Resource Centers for Minority Aging Research (RCMAR). It is a collaborative research, community outreach, and scientist mentoring program based at Wayne State University, Michigan State University, and the University of Michigan. The purpose of the MCUAAAR is to increase and enhance the diversity of the future scientific research workforce, mentor promising new faculty and research scientists from under-represented groups for sustained careers in aging-related behavioral research, and to collaborate with community-based organizations in Detroit and Flint, Michigan. The community-based activities are implemented through the Community Liaison and Recruitment Core (CLRC). The CLRC contributes to the science of recruitment and retention of older African Americans through the unique Participant Resource Pool (PRP). The PRP is a research registry of 1,100 community-dwelling African Americans aged 55 or older that are willing to be invited to participate in research studies. The PRP and community outreach is coordinated out of the Healthier Black Elders Center (HBEC). HBEC aims to target and reduce health disparities through community education and by increasing African American older adults’ participation in research through the PRP. HBEC maintains partnerships with community-based organizations in Detroit and Flint to host on-going health-focused community education events, Lunch & Learns, and disseminates to and translates scientific information for those communities through bi-annual paper newsletters and monthly emails. All HBEC activities are overseen by a sixteen member Community Advisory Board (CAB). CAB members are the decision-makers for all HBEC program activities and function as the gatekeepers for research given that they are charged with reviewing and approving all applications to use the PRP for research recruitment (17).

Since 2016, the Michigan ADRC has been conducting and facilitating a wide range of research studies pertinent to Alzheimer’s disease and related dementias. Including investigators from the state’s three leading research universities (University of Michigan, Michigan State University, Wayne State University), it is part of a national network of ADRCs funded by the National Institute on Aging. The major theme of the Michigan ADRC is to “identify, understand, and modulate the many factors beyond beta-amyloid that contribute to brain dysfunction and neurodegeneration in Alzheimer’s disease and related dementias (ADRD).” The Center’s major areas of emphasis include: capturing the diverse expertise of dementia scientists, shedding light on ADRD heterogeneity, investigating factors contributing to cognitive impairment in underrepresented and underserved communities, identifying and disclosing disease risk and research results, developing non-pharmacological, non-invasive approaches to treatment, and understanding mechanisms of proteinopathies underlying ADRD. Through its Clinical Core, the Michigan ADRC is home to a longitudinal cohort study of older adults both with and without memory impairment, where survey, neuropsychological, neuroimaging, and genetic data are pooled at the National Alzheimer’s Coordinating Center with data from similar studies conducted nationwide at the other ADRCs. The longitudinal cohort currently includes 469 participants (64% female and 33% Black/African American). The Michigan ADRC also hosts a research registry of individuals who have expressed interest in participating in AD-related studies. This registry currently holds 1,183 participants – 25% Black/African American and 64% female – and is accessible to investigators needing assistance with study recruitment and enrollment. The registry has been used to support a wide range of projects including drug trials, non-pharmacological interventions, observational studies, and studies focused on issues including caregiving, driving, financial management, and disclosure of genetic/biomarker results. The Center’s community engagement efforts are led by its Outreach, Recruitment, and Engagement (ORE) Core, whose education and outreach activities promote involvement in Center-sponsored studies and AD research in general. Participants have been recruited from across the state of Michigan, with the majority coming from its southeast sector and having learned of the Center through community outreach efforts in the Detroit metropolitan area.

The University of Michigan Claude D. Pepper Older Americans Independence Center is housed in the Geriatric Center within the Medical School at the University of Michigan. Since 1989, the goal of the Center has been to provide junior faculty training to develop their careers in conducting critical research to address health problems of older adults. The Center’s funding mandate is to assist researchers with relevant education, provide mentoring, and the resources necessary to successfully conduct pilot projects. Thus, the focus historically has been to assist junior researchers in recruiting relevant samples for specific studies. Since the Center’s inception over 2,000 older adults have been recruited into a Center research participant program through community outreach, mailing lists, and enrollment from other Center-supported studies. Current enrollment in the Center registry is maintained at approximately 600 older adults. In recent years, emphasis has been placed on facilitating studies in geographic areas where diverse groups of older adults reside so that participation does not require travel to Ann Arbor. Facilities for data collection and sites for recruitment outreach from local senior centers, health fairs, and various organizations have been utilized. Additionally, because many of the studies supported by the Center entail extensive medical screening, training and support has been provided in the use of and recruitment from electronic health records with particular emphasis on techniques to ensure diverse samples. More broadly, the focus has been on ensuring that older adults from diverse populations are represented in research and that new researchers are being trained to conduct research that promotes the health and well-being of all older adults.

### Collaboration goals

Confronted with the challenges of the COVID-19 pandemic, we decided to develop a learning community among the four Michigan NIH-funded Centers to share lessons learned and provide a supportive environment to staff members. We found that the programmatic focus of our community organization partners shifted to address the immediate need of the communities they serve. To strengthen our relationship with each community and enhance the trust as a source of scientific information, we needed to effectively meet the programmatic shift of our community organization partners and support their efforts to address the immediate needs of each community. This represented unprecedented challenges and extraordinary opportunities to adapt our program activities to meet community needs while aligning the required changes to the Centers’ research goals and expected outcomes. Thus, the sudden and unprecedented circumstances, caused by the COVID-19 Pandemic, called for refocusing efforts to assess the needs of community members and partners to sustain engagement and provide support to those in need, especially older adults. Below, we highlight strategies used by our Centers (Table 1). We found that sharing lessons learned about implemented strategies helped us adopt changes to better serve community partners and individuals.

**Table 1:** Michigan National Institutes of Health funded Centers.

Center	Mission	Core outreach
Michigan Center for Contextual Factors in Alzheimer’s Disease (MCCFAD)	MCCFAD focuses on generating knowledge concerning the contexts of Alzheimer’s disease and related dementias (ADRD) by elucidating sociocultural, economic and behavioral contributors to and consequences of health disparities, specifically among the Middle/Eastern/Arab Americans in the metropolitan Detroit area and Latino communities in the Grand Rapids Metropolitan area	MCCFAD’s Community Liaison and Recruitment Core (CLRC) uses a community-based partnership research approach to develop and disseminate culturally tailored health education programs to reduce risk of ADRD, encourage research participation by inviting community members to become part of a Research Volunteer Directory, and enhance participation in the healthcare system
Michigan Center for Urban African American Aging Research (MCUAAAR)	MCUAAAR is a collaborative research, community outreach, and faculty mentoring program based at Wayne State University (WSU), Michigan State University (MSU), and the University of Michigan (U-M). One of MCUAAAR’s goals is to address and reduce health disparities through increasing older Black adults’ participation in research and providing pertinent health information to the Flint and Detroit communities	Our community-based activities are implemented through the Community Liaison and Recruitment Core (CLRC). The CLRC contributes to the science of recruitment and retention of older African Americans through the unique research registry, the Participant Resource Pool (PRP). The PRP maintains partnerships with community-based organizations in Detroit and Flint, and disseminates to and translates scientific information for those communities through print materials and Lunch & Learn events. The PRP and community outreach efforts are coordinated out of the Healthier Black Elders Center (HBEC)
Michigan Alzheimer’s Disease Research Center (Michigan ADRC)	Michigan ADRC conducts and facilitates a wide range of research topics pertinent to Alzheimer’s disease and related dementias. Including investigators from the state’s three leading research universities (U-M, MSU, WSU), it is part of a national network of ADRCs funded by the National Institute on Aging	The Center’s community engagement efforts are led by its Outreach, Recruitment, & Engagement (ORE) Core, whose education and outreach activities promote involvement in Center-sponsored studies and AD research in general. Participants have been recruited from across the state of Michigan, with the majority coming from its southeast sector. African Americans are a population of particular interest, representing approximately 40% of the Center’s registry participants. Most of these individuals have learned of the Center through community outreach efforts in the Detroit metropolitan area
University of Michigan Claude D. Pepper Older Americans Independence Center (Pepper Center)	Pepper Center is housed in the Geriatric Center within the Medical School. The goal of the center is to provide training to junior faculty to develop their careers in conducting critical research in health problems of older adults. Our main objectives are to assist researchers with mentoring and relevant education and provide the resources necessary to conduct pilot projects	At the Pepper Center, our focus historically has been to assist in recruiting participants for specific studies. More broadly the focus has been on ensuring that older adults are represented in research and that new researchers are being trained to do research that promotes the health and well-being of older adults

## Methods

Starting in April 2021, the four NIH-funded Centers organized a 90 min monthly virtual meeting that included Co-Leads and staff of the respective community outreach cores. The structure of this virtual meetings was:

*Meeting participants*: Center’s director and Community Liaison and Recruitment Cores’ Co-Leads and staff of each Center. A Core’s Co-Lead (MCCFAD) sent the agenda and moderated the meeting.

*Schedule*: Starting on April 2021, a monthly meeting was scheduled. On April 2022 the virtual meeting was changed to quarterly. A Core’s administrator sent calendar invites, containing the zoom link for the virtual meeting.

*Agenda*: An agenda was developed with specific topics decided by the members at the end of each meeting. Meeting agendas were sent ahead of time listing discussion topics, and all meeting materials (i.e., agendas, video recordings, minutes) were uploaded to a shared folder. The topics were based on pending discussion or to report progress on specific Core’s activities. Core presented their best practices to community outreach and engagement approaches, experience pivoting to virtual engagement, Community Advisory Board experiences, and recruitment efforts. Discussion about implementation processes, promotion strategies for community events, logistics, virtual platforms, and digital resources (e.g., surveys, evaluations) allowed the members to understand the nuances of each approach. Feedback and recommendations from community partners and advisory board members were also discussed as a tool to assess the approaches and strategies used.

*Assessment*: Events and strategies were assessed quantitatively and qualitatively using the meeting video recordings and meeting minutes. Quantitative measures included number of participants, views of digital resources, and number of community members contacted. Qualitative assessment included reviewing each core’s activities and strategies for similarities and differences and the comments and feedback from community partners and advisory board members. The conversations throughout our monthly meetings led to the recompilation of best practice approaches and strategies discussed. Here, we synthesized those conversations to provide key insights about successes and challenges using community-strategies to engage and retain vulnerable populations during the pandemic in research.

Terms used in the text:

1. Researchers and staff – refer to staff at each Center that participated in the meetings, research, and community activities.
2. Health professionals – refer to university and center-affiliated health professionals, including physician scientists and nurses.
3. Research Participants – refer to individuals recruited and participating in ongoing research interventions.
4. Community members – refer to individuals that participated in community events, such as learning and education events.
5. Learning and education events – refer to activities programmed at community sites.

## Results

### Community-based strategies to alleviate the burden of the COVID-19 pandemic on vulnerable communities in Michigan

#### Strategies to alleviate the burden of the COVID-19 pandemic

Education about COVID-19 and how to reduce the risk of infection was an important opportunity to strengthen the relationship with community organizations and enhance our presence as a trusted source of scientific information within the community. For example, in April 2020, MCCFAD established relationships with local radio stations, news outlets and community-based organizations allowing us to organize panels with physicians in West Michigan, in both Spanish and English. During these interventions, we discussed topics such as how a coronavirus spreads, how to reduce the risk of infection, what are the most prevalent symptoms, which comorbidities were associated with higher risk of severe outcomes, which groups are at higher risk of infection and how to protect the family when one member is positive for COVID-19. It originally appeared as a challenge to align this new education effort related to viral infection with established educational programs directed to reduce the risk of ADRD and promote participation in research. However, as more research was published and information on SARS-CoV-2 became available, we were able to align the information on COVID-19 with established learning objectives related to reducing the risk of ADRD. Our presentation on the importance of preventing or properly treating metabolic disorders known to be risk factors for ADRD was tied to its association with severe outcomes of COVID-19. Neuronal manifestations (i.e., anosmia and dysgeusia) and encephalopathy associated with COVID-19 allowed us to explain the function of the central nervous system, define stroke, how brain inflammation leads to damage, associate brain structure and function, the importance of oxygen to the brain, explain what brain fog is, and the relationship between COVID-19, traumatic brain injury and risk of ADRD.

Throughout the evolution of the COVID-19 pandemic, MCCFAD also adapted the educational programs to include the importance of vaccination, contributing to the educational programs of community organizations and public service of media outlets. Community partners requested that we participate in panels and presentations to the community about the newly developed topics. The partner organizations included the Hispanic Center of Western Michigan, Detroit Hispanic Development Corporation, Latino Community Coalition, Spectrum Health Cost Health Team, Latin Americans United For Progress, among others. The number of participants in the virtual presentations varied from 50 to 10 per event, which depended on the community partner’s promotion of the event and organization members.

Similarly, MCUAAAR, the Healthier Black Elders Center (HBEC), leveraged its long-standing relationship with the Detroit community as a trusted resource for health information. It was imperative to continually print updated COVID information in the program newsletter mailed to all 1,100 registry members (e.g., cleaning vs. disinfecting, how do COVID-19 vaccines work in the body, recognizing signs of depression), and include personal testimonies of HBEC member’s experience of COVID-19. During the holiday season in 2020, HBEC mailed a letter to all registry members with a resource guide for mental health services, COVID-19 testing sites, food assistance, transportation, utilities and financial support. The letter also included a guide on how to stay safe during the holidays visiting family and friends. In addition to printed health information, HBEC hosted several virtual Lunch & Learn events with health professionals to present information on COVID-19 and the vaccines. Partnerships with AARP-MI, university-affiliated health professionals, Detroit Area Agency on Aging, St. Patrick Senior Center, Hannan Foundation, Alzheimer’s Association, Eastside Community Network, and many local organizations supported and further leveraged our outreach efforts. The attendance to virtual events from September 2020 through December 2021 totaled 12,990 older adults. The main challenge was to keep up with the rapidly increasing literature on COVID-19 and disseminate the new knowledge in a way that was accessible to everyone in the community, while maintaining our established program identity within the community.

#### Social support strategies to enhance community partnerships

Supporting MCCFAD’s community organization partners during the earlier days of the pandemic also involved attention to ADRD caregiving challenges. For example, a COVID-19 tip sheet was created given that uncertainty and tension resulting from the COVID-19 pandemic likely added some or a lot of distress to providing care. We recognized that providing care to a family member with Alzheimer’s disease or a related dementia (ADRD) does not stop because of COVID-19, and so sought to create a list of tips for caregivers. This tip sheet was translated into Arabic and disseminated to our community partners as a resource. MCCFAD also recorded virtual health education programs in Spanish and English, done in collaboration with Grand Rapids Public Library (GRPL), and became an archive of information available to all community members. The 20-min videos are now accessible *via* the GRPL online collection and became an added resource to share content with community members.

Likewise, MCUAAAR developed a telephone outreach project during the first year of the COVID-19 pandemic to reach active members in the research registry (18). The purpose of the project was to decrease social isolation, broker resources, and stay in touch with registry members. The callers conducted a telephone survey with questions on wellness, strengths, challenges, access to services (healthcare, technology), social and family life, mental health, and identified any unmet needs. At the end of the project, over 900 people were contacted, 557 surveys were completed, and staff connected participants to various social services, such as mental health services, masks, grocery delivery, financial services, and caregiver support. A vital component to the project was the monthly debrief meetings with the project team callers. The purpose of the debrief meetings was to check-in with the caller’s wellbeing and to provide feedback on successes and challenges conducting calls. It was critical to hold these frequent meetings to provide support as needed to the callers throughout the project as they collected information on how registry members were experiencing the pandemic while also living through the pandemic themselves (15).

The Michigan ADRC conducted similar activities, initially contacting research participants (*n* = 407) in the Center’s longitudinal cohort, the University of Michigan Memory and Aging Project (UM-MAP), to check in and provide needed resources and support. Information on available local resources (e.g., groceries, masks, testing sites, mental health support) was gathered in preparation for these outreach calls and shared with recruitment coordinators making calls. Additionally, website and social media content was updated to include information about these critical resources as well as local programs and resources available to those in need.

#### Innovative social media events to adapt to a virtual world

Finally, all four Centers halted all in-person events and instead created virtual events. Innovations were identified to live stream on multiple social platforms including Facebook and YouTube, allowing events to be recorded and available for future viewing. Yet, success in connecting with the community virtually varied by center. For example, before the HBEC virtual Lunch and Learn series began, the printed HBEC newsletter included a full-page step-by-step guide with images instructing members on how to join Zoom meetings by phone or computer. Many registry members were able to use this guide to assist them in joining HBEC programs, but also to participate in other Zoom meetings with family, friends, church groups, exercise classes, and more. Average attendance of virtual Lunch and Learns ranged from 20 to 50 older adults, with half joining by telephone and the other by computer. All Lunch and Learns were recorded and uploaded on the program website and distributed in monthly emails to registry members.

Michigan ADRC Dementia Lecture Series presentations, including topics of drug trials, brain donation, and dementia risk reduction, were adjusted to be offered virtually on a monthly basis instead of in-person at the U-M Detroit Center. Moving online increased the average attendance per event from 55 attendees to 120 attendees, although this increase was driven primarily by an increase in health professional audience members rather than community dwelling older adults. Other existing Michigan ADRC programs such as caregiver wellness programming (e.g., mindfulness-based stress reduction groups) and Lewy body dementia (LBD) support groups successfully transitioned to online offerings and the Center created new virtual programs throughout the pandemic (e.g., a “Mitten Minds” Education Series for newly diagnosed persons with dementia and their family members) to meet the unique set of challenges for individuals living with dementia and their caregivers unable to leave their homes. Nevertheless, in general, the transition to virtual activities led to reduced participation from community dwelling adults with less technology proficiency or internet access.

Despite our collective efforts, success to increase participation in virtual events varied depending on population demographics. Further, the transition from in-person to virtual presentations brought another set of challenges such as selection of the best day of the week, time of day to not compete with household chores and in-home schoolwork, as well as technology use. For example, even though different days and times were tried, participation in virtual presentations organized by MCCFAD in collaboration with partner community organizations fluctuated between 5 and no more than 20 people. In contrast, Michigan ADRC Dementia Lecture Series experienced an increase in attendees. The content and audiences per virtual event contributed to the differences in participation. The main gain in the transition to virtual event and use of social media platforms was to develop strategies that, after the ease of restrictions, allowed to incorporate the virtual option to in-person events making them more accessible to community members to engage in-real time or archive the event for future viewing.

### Community engagement strategies to retain research participants and enhance research participation in Michigan during the COVID-19 pandemic

#### Strategies to enhance research participation during the COVID-19 pandemic

An important goal of our Centers is to enhance the participation of underrepresented groups in research and the healthcare system. The COVID-19 pandemic uncovered the healthcare inequities and barriers that affect underserved communities. The lack of availability of COVID-19 testing in combination with the existing lack of access among underserved communities to healthcare demanded that we demonstrate with actions as to how we could minimize the effects of healthcare inequities.

One example of an action undertaken was MCCFAD’s effort to organize community leaders and use established relationships with healthcare providers to bring to the forefront the healthcare needs of the Latino community in Grand Rapids, West Michigan. These interactions led to identifying barriers at testing sites, such as language needs, service hours and locations, making healthcare providers and government agencies more aware of the need for culturally sensitive messaging and implementation of services. As a result, we were included in the Kent County Health Department’s weekly Community COVID-19 Vaccine Planning meetings as representatives and advocates of the Latino community in West Michigan. At the same time, the Department of Translational Neuroscience at Michigan State University developed a cost-effective COVID-19 test based on self-collections using a nasal swab as well as saliva (19). This represented an opportunity to validate the new test using samples from the community, which at the same time allowed the COVID-19 test to be available to underserved communities. Validating the new COVID-19 test in the community also allowed us to expose community members to relevant and opportune research with clear and immediate implications. After IRB approval, in collaboration with MCCFAD’s community partners in the Grand Rapids area, we consented and collected samples from community members from April 2020 to December 2020. During this period, we consented 224 participants of which 73% were Latinos. Further, Centers’ members signed up as research participants of what was at the time experimental vaccines against COVID-19. This allowed Centers’ members to serve as role models for participation in clinical trials and provide first-hand accounts on how the vaccine trials were being conducted. The combination of vaccine education and advocacy work led to the establishment of vaccine clinics at sites within underserved communities. However, there remain barriers to accessing the healthcare system and vaccine hesitancy among members of underserved communities, including trust issues, that need to be addressed.

While new participant enrollment was a challenge during the pandemic, Michigan ADRC’s existing UM-MAP participants were able to complete telephone or computer visits to remain active in this longitudinal research study at a time when University protocols prohibited in-person research activities. The Michigan ADRC’s efforts to retain its participants relied heavily on Center staff who had already developed longstanding relationships with Center participants. For example, Dr. Edna Rose, a PhD level nurse with decades of community outreach experience, completed the majority of the 271 telephone visits with participants during this time and dedicated much of her time to volunteering at vaccination sites around the city of Detroit where she was able to engage with active research participants in person. While at public testing sites, she shared helpful educational information about addressing memory and thinking changes, caregiver resources, and ongoing Center research opportunities with any of the community members in attendance who were interested, in addition to sharing information on COVID-19 vaccine safety with anyone who had questions or hesitations about the vaccine.

#### Community engagement strategies

Another example of an innovative response to the pandemic and the barriers it created for community engagement was another outcome of the MCUAAAR’s Telephone Outreach Project. This project allowed for needs assessments among community members during the pandemic as well as facilitated retention of participants in the registry of potential research participants. Many registry members expressed gratitude for the educational Lunch & Learn events but reported increased feelings of loneliness and isolation. This feedback led to the launch of a new weekly program, Party Line. Party Line was given its name by an HBEC staff member’s mother, to invoke the spirit of the actual “party line” phone calls that she remembered from her youth. It is a virtual social group co-hosted by MCUAAAR’s Community Outreach Coordinator and a CAB member that invites older adults to socialize and discuss any topic of their choice. Party Line has been so successful, members requested it continue past the original timeline of ending in 2021. In addition to creating new friendships, members self-coordinated outdoor meetups outside the virtual Party Line meetings. In 2021 and 2022, a total of 464 older adults attended Party Line. Majority are existing members of the registry, however three new members were recruited, and it is still running to date. Party Line is one example of how HBEC adapted program activities to meet the needs of registry members that fell outside the center aims yet still contributed to retention and recruiting new members to the registry. Similarly, MCCFAD increased its social media presence, with more frequent use of Facebook TikTok and Instagram to keep the community informed. Newly developed online forms became a new tool in the arsenal to recruit community members into the research volunteer directory. However, the number of enrolled community members using the online forms did not reach the successes achieved when done at or after in-person activities.

The UM Pepper Center converted from in-person research visits to telephone visits for three on-going longitudinal studies to maintain engagement of over 600 participants. The calls also focused on participants’ safety and availability of support during the pandemic. New recruitment to one of these studies was converted to telephone recruitment and verbal consent consistent with institutional restrictions on in-person research contacts. Early in the pandemic, the Pepper Center joined a Medical Center team to ensure that older adults were represented in two trials of COVID vaccine safety and efficacy. Email and telephone calls were made to introduce the trials and address any concerns of approximately 500 potential participants. The focus of these calls was, in part, to educate community members on the conduct of clinical trials and the importance of diverse participation to ensure that findings are applicable to all older adults. Pepper Center staff completed trial eligibility screening and enrollment tasks for a large cohort of older adults. The Pepper Center also introduced the availability of the vaccine trials in a virtual session focused on educating older adults on COVID vaccines as part of the University of Michigan Osher Lifelong Learning Institute programming.

The strategies and efforts used to maintain engagement with research participants and those enrolled in research directories or registries allowed the staff to have closer communication with community members, even though the recruitment of new participants was limited. Research participants and those enrolled in our directories/registries expressed their gratitude for reaching out during the time when older adults were feeling isolated due to social distancing policies. Additionally, staff members used the opportunity to sign up for the ongoing vaccine clinical trials, allowing them to acquire experiences from the perspective of a research participant while serving as a role model to community members. The collective lessons learned, established partnerships and community advocacy efforts also enabled community-based organizations to successfully acquire state and federal funding earmarked to enhanced vaccination, especially within underserved communities (20). At the end, the community partnerships, advocacy work and engagement during isolation brough new resources to the community and provided an opportunity to be closer to community members.

## Discussion

The lessons learned throughout the COVID-19 pandemic of different groups in government and academic settings have been compiled in different published articles (1–7). The common denominator in all the shared experiences was the importance of listening to the community and effectively responding to their needs and concerns (1–7). Additionally, some reports discussed how specific institutes or programs supported staff members through the stress and zoom fatigue generated throughout the pandemic (21–23). These efforts were conducted principally by an academic program or collaborative programs within the same institution. In contrast, here we described how four NIH-funded centers (distributed across three different academic institutions in Michigan), dedicated their efforts to enhancing the research participation of underrepresented groups to advancing the science of aging by joining together during this difficult time to learn from each other and implement identified best practices in-real time. Increased interactions and creation of a learning community among Centers dedicated to service and research was an important positive development from the COVID-19 pandemic. As a result, Centers learned new approaches from one another on how to manage stress generated among community outreach staff due to institutional shutdown and suspension of all in-person activities. The staff needed to adapt to virtual meetings and maintain contact with community partners. Working from home while having children engaged in virtual classes represented an added stress (21). This unprecedented work-family merging situation was taking place during increased job insecurity due to the imposed shutdown on in-person research activities. Zoom fatigue was adding to the frustration of developing new strategies perceived to be temporary, as reported by others (22, 23). Flexibility, compassion, and effective leadership became tools to reduce stress and promote innovation while recognizing the difficult situation that everyone was facing.

Another gain was the understanding that team leaders needed to first reaffirm institutional commitment to protecting the employment of the Centers’ staff members and then adapt to changing perspectives. Of particular importance was the development of new approaches to job duties that were driven by the pandemic situation. The four Centers’ synergistic activities led to the conclusion that pandemic driven changes were not temporary. As the evolving pandemic continued its course and staff adapted to new approaches, it became clear that the new tools and strategies arising from pandemic constraints would remain. Virtual activities became hybrid activities with the easing of restrictions, providing a sense of enhanced inclusion and equity for all community members. The staff’s efforts to develop and implement online forms and recruitment strategies to enroll community members into the Centers’ research volunteer registries or directories resulted in supplementary and inclusive outreach alternatives that are still used following reinstatement of in-person activities. Therefore, the effort and products generated during the height of the pandemic became permanent tools used in community outreach and engagement.

Notably, the four Centers shared information and addressed challenges regarding institutional mandates and requirements to support one another’s goals while benefiting from pooled experiences. During these difficult times, our Centers became more connected through established monthly meetings where lessons learned were shared and everyone had opportunities to ask questions and learn from each other’s successes and challenges. These monthly meetings also became brainstorming sessions to address specific challenges all Centers were facing regarding recruitment, retention and outreach activities beyond the COVID-19 pandemic. The informal format allows everyone the opportunity to share their experiences in a safe and supportive environment, serving as respite to Centers’ leaders and staff. We shared details about specific challenges such as promoting and enhancing participation in virtual events. After discussing specific strategies, we were able to report back on our successes and remaining challenges. These meetings also allowed us to be empathetic about reduced participation in a virtual event or enrollment into the registry/directory despite enormous efforts, assuring everyone that we were, indeed, dealing with unprecedented circumstances. Everyone agreed that the supportive and productive environment generated at these monthly meetings strengthened our unique Center partnerships. Moreover, these meetings allowed us to speedily address challenges and understand community needs to enhance enrollment and participation in research.

Importantly, we were able to adapt our educational material from healthy aging and risk factors in Alzheimer’s disease and related dementias to incorporate COVID-19 and its impact on the brain. COVID-19 symptomatology included loss of smell, taste, joint and muscle pain/weakness and brain fog, among others (24, 25). Blood biomarkers associated with brain injury were also identified among individuals with COVID-19 (26). For the Centers focused on Alzheimer’s disease and related dementias, these facts allowed us to talk about brain health within the context of the COVID-19 pandemic and why it has been considered as a new risk factor for Alzheimer’s disease and related dementias. Unique opportunities to leverage community partnerships to bring COVID-19 testing within underserved communities in Latino communities strengthened relationships with community organizations and allowed provision of a service not accessible to all. A limitation was that this effort took place only in West Michigan as part of MCCFAD’s Community Liaison and Research Core. Nevertheless, the experiences gathered during this process were shared with other Centers and served as foundation to develop new ongoing projects in partnership with community organizations. Hence, we illustrate how and where the developed learning community among four NIH-funded Centers through the COVID-19 pandemic introduced new pathways to engaging with and encouraging research participation among older adults and those in underrepresented communities.

## Conclusion

In sum, the COVID-19 pandemic renewed all four Centers’ commitment to community service. To achieve our goals of promoting more diverse participation in research, we affirmed the importance of taking into consideration the needs of the communities to be included in research. The identified needs of our partner community-based organizations and community members during these challenging times served as the foundation that guided the initiatives and activities outlined above. By adapting our educational activities to address community needs, we valued serving as a trusted resource of information in a society forced to dissect differences of opinions regarding important health concerns. Through these interactions, we confirmed that rather than diluting our outcome-based research goals, emphasis on community service and becoming a trusted resource of information are efforts that bring us infinitely closer to our research goals.

## Data availability statement

The original contributions presented in the study are included in the article/supplementary material, further inquiries can be directed to the corresponding author/s.

## Ethics statement

The studies involving human participants were reviewed and approved by Health Sciences and Behavioral Sciences Institutional Review Board (IRB-HSBS), University of Michigan; MSU Human Research Protection Program. The patients/participants provided their written informed consent to participate in this study.

## Author contributions

IV led the conceptualization and development of the manuscript. KA, VR, RG, JR, and LN collaborated on the conceptualization of the community case study and proofread the manuscript. All authors contributed to the article and approved the submitted version.

## Funding

This publication is a product of the work conducted at all four Centers. The Centers are funded, in part, by the National Institute on Aging: MCCFAD P30AG059300, MCUAAAR P30AG015281, Michigan ADRC P30AG072931 and Pepper Center P30AG024824.

## Conflict of interest

The authors declare that the research was conducted in the absence of any commercial or financial relationships that could be construed as a potential conflict of interest.

## Publisher’s note

All claims expressed in this article are solely those of the authors and do not necessarily represent those of their affiliated organizations, or those of the publisher, the editors and the reviewers. Any product that may be evaluated in this article, or claim that may be made by its manufacturer, is not guaranteed or endorsed by the publisher.
